# Neurological symptoms in acute COVID-19 infected patients: A survey among Italian physicians

**DOI:** 10.1371/journal.pone.0238159

**Published:** 2020-09-11

**Authors:** Laura Campiglio, Alberto Priori

**Affiliations:** Department of Health Sciences, “Aldo Ravelli” Center for Experimental Brain Therapeutics and Clinical Neurology Unit, University of Milan, Italy, San Paolo University Hospital, ASST Santi Paolo e Carlo, Milan, Italy; Chinese Academy of Medical Sciences and Peking Union Medical College, CHINA

## Abstract

**Background:**

COVID-19 is a pandemic disease and questions rise about the coronavirus 2 (Sars-CoV-2) effect on nervous system. This involvement could help explaining the pathogenesis of this condition and lead to novel therapeutic approaches.

**Objective:**

To assess the occurrence of neurological symptoms in COVID-19 patients during the Italian pandemic outbreak, as reported by physicians.

**Materials and methods:**

In the early days of pandemic emergence we developed an online survey open to all Italian clinicians involved in the diagnosis and management of COVID-19 patients. The survey was structured in three sections, with nine different items concerning the presence of different specific clinical abnormalities. Each item was graded from “absent” to “severe” in a 4-point Likert’s scale.

**Main outcomes and measures:**

Likert’s scale data were analyzed by studying the distribution of responses by using medians and bar charts-relative frequencies. Also, in order to analyze differences in symptoms findings depending on the group of specialty, Likert’s scale data were combined into two nominal categories (“absent”/“low” and “moderate”/”high”/”) and a contingency table chi-square test was used.

**Results:**

126 physicians of 9 different medical specialties, from 10 regions of Italy, filled the online survey. The results show that 87.3% of practitioners reported neurological symptoms. In most cases these were mild and non-specific, but they were severe in a minority of patients. The most common symptoms observed were headache, myalgia and taste and smell abnormalities. Whilst there was no difference between neurologists and non-neurologists, we found that experienced clinicians (defined as clinicians that evaluated more than 30 patients) reported neurological symptoms more frequently than non-expert.

**Conclusions:**

Neurological symptoms have frequently been ported during the Italian COVID-19 pandemic, and thus should be monitored for all affected patients. Whilst some of the disturbances reported may be non-specific and common to other infectious diseases, smell and taste abnormalities might indicate nervous system as entry door for SARS-CoV-2 virus. This interpretation should promote research trials to avoid nervous system involvement.

## Introduction

In March 2020 Italy became the second most affected country in the world and death toll overtook those in China. Symptoms of COVID-19 include respiratory illness, fever, dry cough and dyspnea [1, 2]. In severe cases, patients have interstitial pneumonia with acute respiratory distress syndrome (ARDS) and require mechanical ventilation [1, 2]. There is a growing evidence that SARS-CoV-2 can involve organs other than the lung, including the nervous system [3, 4]. The most common neurological symptoms reported are headache, anosmia and ageusia [5–7]. Other common symptoms include stroke, impairment of consciousness, seizure, and encephalopathy [8–10]. Some authors suggested that SARS-CoV-2 neurotropism could contribute to the severity of respiratory failure [11, 12]. Netland et al. reported the involvement of cardiorespiratory centers in the medulla of transgenic mice for the SARS-CoV receptor (human angiotensin-converting enzyme 2) [13]. Because SARS-CoV-2 shows highly homological sequence with SARS‐CoV they can share the same neurotropism [14].

This study aimed to rapidly get an insight into the occurrence of neurological manifestations of the rising epidemic outbreak of COVID-19 disease in Italy by a structured web-based, on-line survey among the Italian physicians involved in the management of acute patients. The data may be useful for other countries that are going to face this dramatic emergency.

## Materials and methods

### Study design and participants

Medical doctors were invited to fill the on-line survey through the Italian medical web community and different social media (WhatsApp, Facebook, Instagram, Websites). 126 colleagues replied in five weeks. The online survey was administered via Google form and was organized in 3 sections:

- Section 1 assessed medical specialty, current geographic region of work and number of cases encountered (<10, 10–30, 30–50, >50 cases)
- Section 2 estimated the frequency of general symptoms and laboratory findings in COVID-19, rated on a 4-point Likert scale from 1 “Never” to “Always”
- Section 3 estimated the frequency of neurological symptoms in COVID-19 patients. The first question addressed the overall presence of neurological symptoms in patients. The second one provided a set of representative symptoms organized in multiple choice grid: each symptom was rated on a 4-point Likert scale from 1 “Never” to 4 “Always”. A 4-point scale was preferred among 5-point scale to avoid neutral responses. In the last item of the survey participants were asked if neurological symptoms were supposed to be “incidental”, “probably correlated” or “directly caused” by SARS-Cov-2.

The full survey is available in Supplemental Materials.

According to the Italian regulation concerning surveys for physicians, the study was not required to be approved by ethical committee. The Alberta Research Ethics Community Consensus Initiative (ARECCI) ethic screening tool was also used to assess and address the ethical dimension of our study: the score was 0, corresponding to a “minimal risk” [15]. In particular: 1) survey data were completely anonymous with no personal information collected; 2) the data were not sensitive or confidential in nature and the issues researched were not likely to upset or disturb participants; 3) participants were not recruited from vulnerable or dependent groups and they get no benefit from participation. Finally, because the study was on voluntary basis, the act of completing the survey was considered an indication of the respondent’s consent to participate. The full results of the ARECCI ethic screening tool are available in Supplemental Materials.

### Data analysis

The survey was developed on the basis of systematic literature review and through discussions with clinicians. We mainly focused on the estimated frequency of a broad list of neurological symptoms to address further studies. Progression to each next question was not possible before answers to all the preceding questions had been registered. The pilot phase indicated that completing the survey took approximately five minutes.

The Likert’s scale data were evaluated analyzing the distribution of responses by using medians and bar charts-relative frequencies. To test the hypothesis that 1) there were no significant differences in neurological symptoms recognition between neurologists and non-neurologists and 2) there would be no difference in the estimated frequency of neurological symptoms between expert and non-expert physicians (defining “expert” physicians that evaluated more than 30 patients) data were combined into two nominal categories (“absent”/”low” and “moderate”/”high”) a contingency table chi-square test was used.

## Results

Replied to the survey 61.1% neurologists, 13.5% specialists in internal medicine and 25.4% physicians from other medical specialties: infectious disease (7.9%), lung disease (2.3%), emergency physician (3.1%) and anesthesiology (0.8%). The majority of participants was therefore not a neurologist. 65.8% surveys were from Lombardy, the area with highest number of patients in Italy. The number of patients evaluated was < 10 for 36.5% of clinicians; between 10 and 30 for 19.0% of them; between 30 and 50 for 19.0% of them and > 50 for 25.4%. Hence, our sample of physicians evaluated a number of patients estimated approximately between 2600 and 4200.

**Fig 1:**
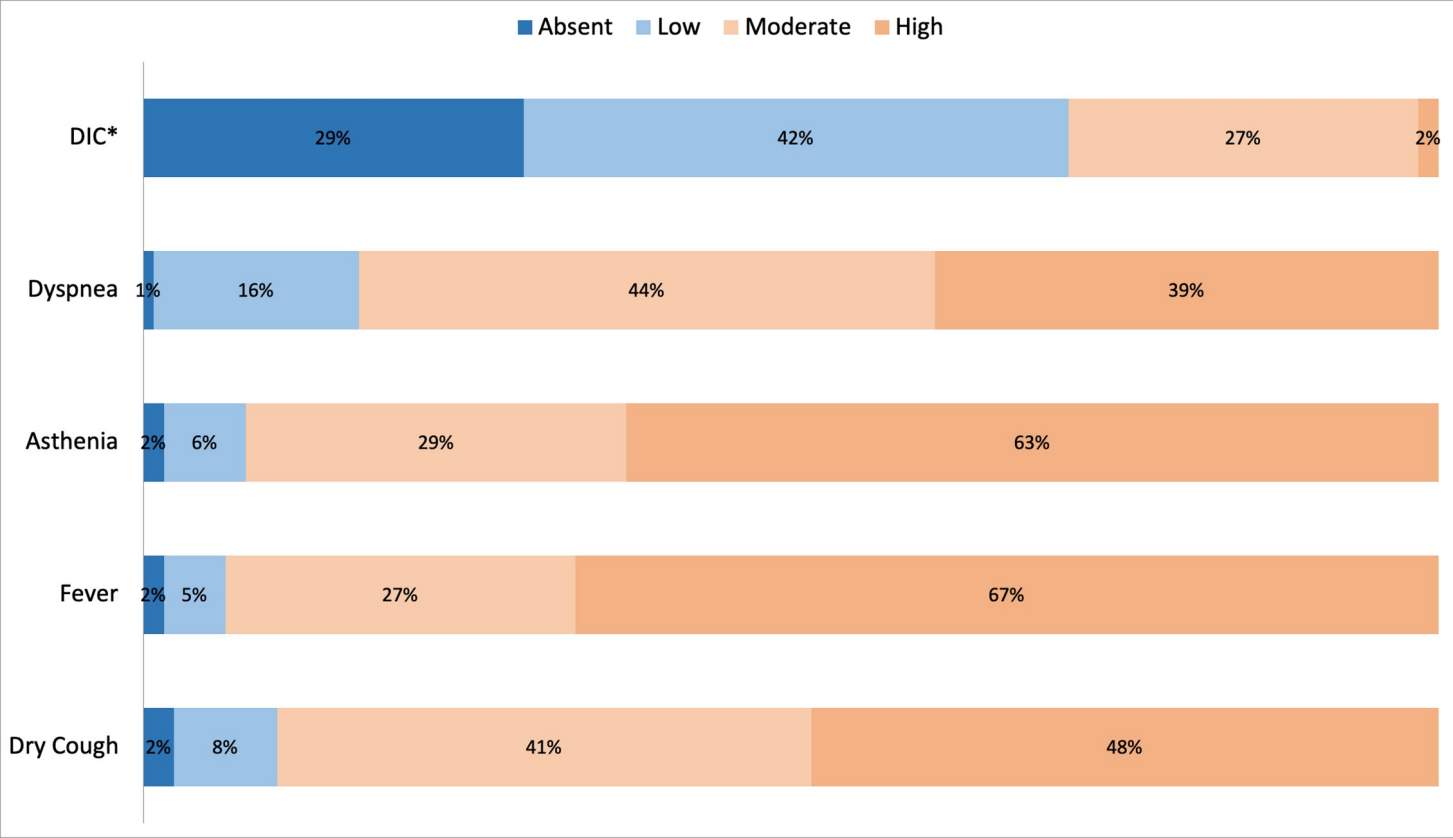
Summary of reply distribution to Question 4: “Evaluate the frequency of these symptoms in COVID-19 patients”. *DIC = disseminated intravascular coagulopathy”.

**Fig 2:**
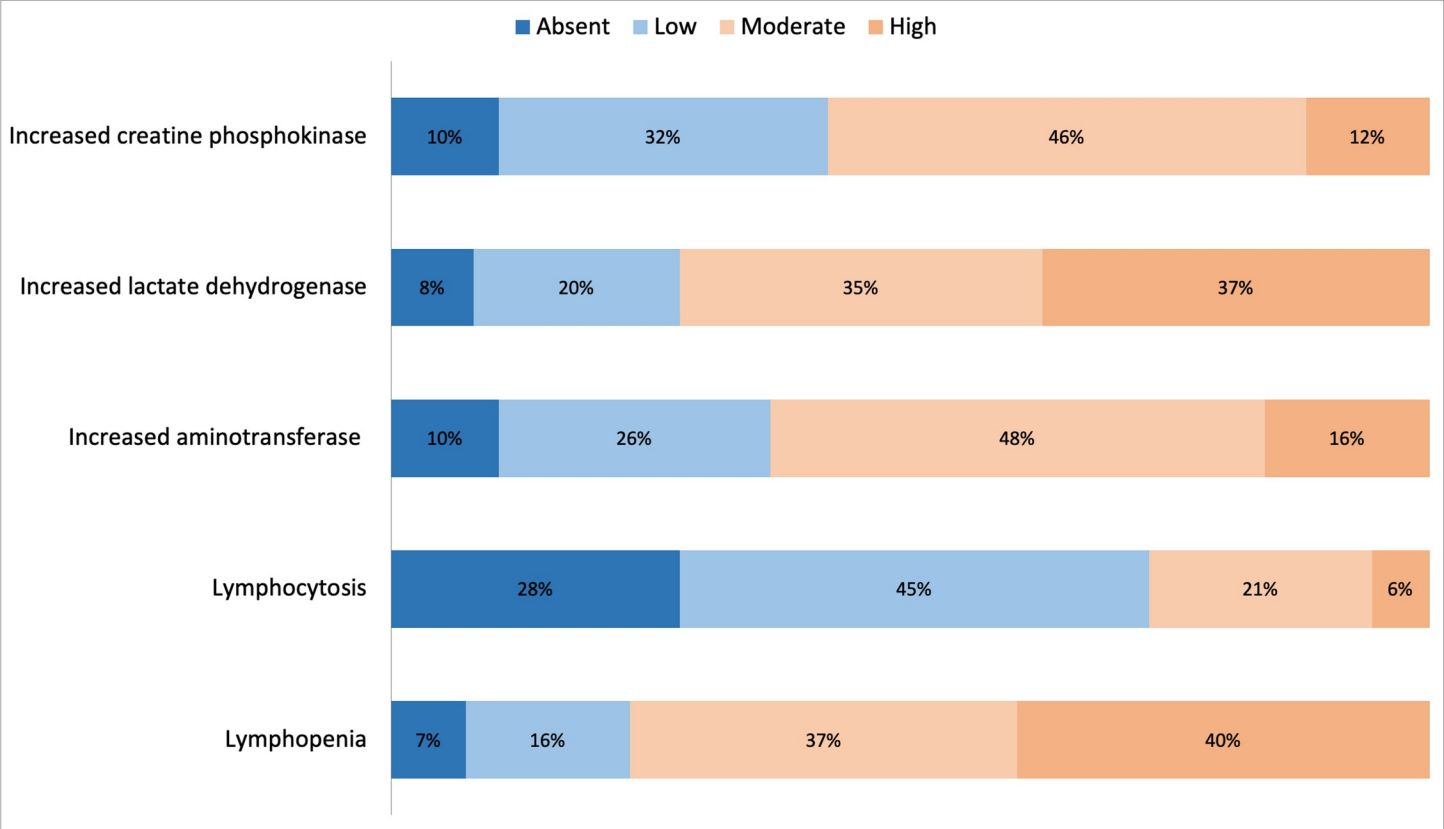
Reply distribution to Question 5: “Evaluate the frequency of these laboratory findings in COVID-19 patients”.

Non-neurological symptoms and laboratory data are consistent with those previously reported thus providing a control variable of the reliability of the survey (Figs 1 and 2) [1, 2].

**Fig 3:**
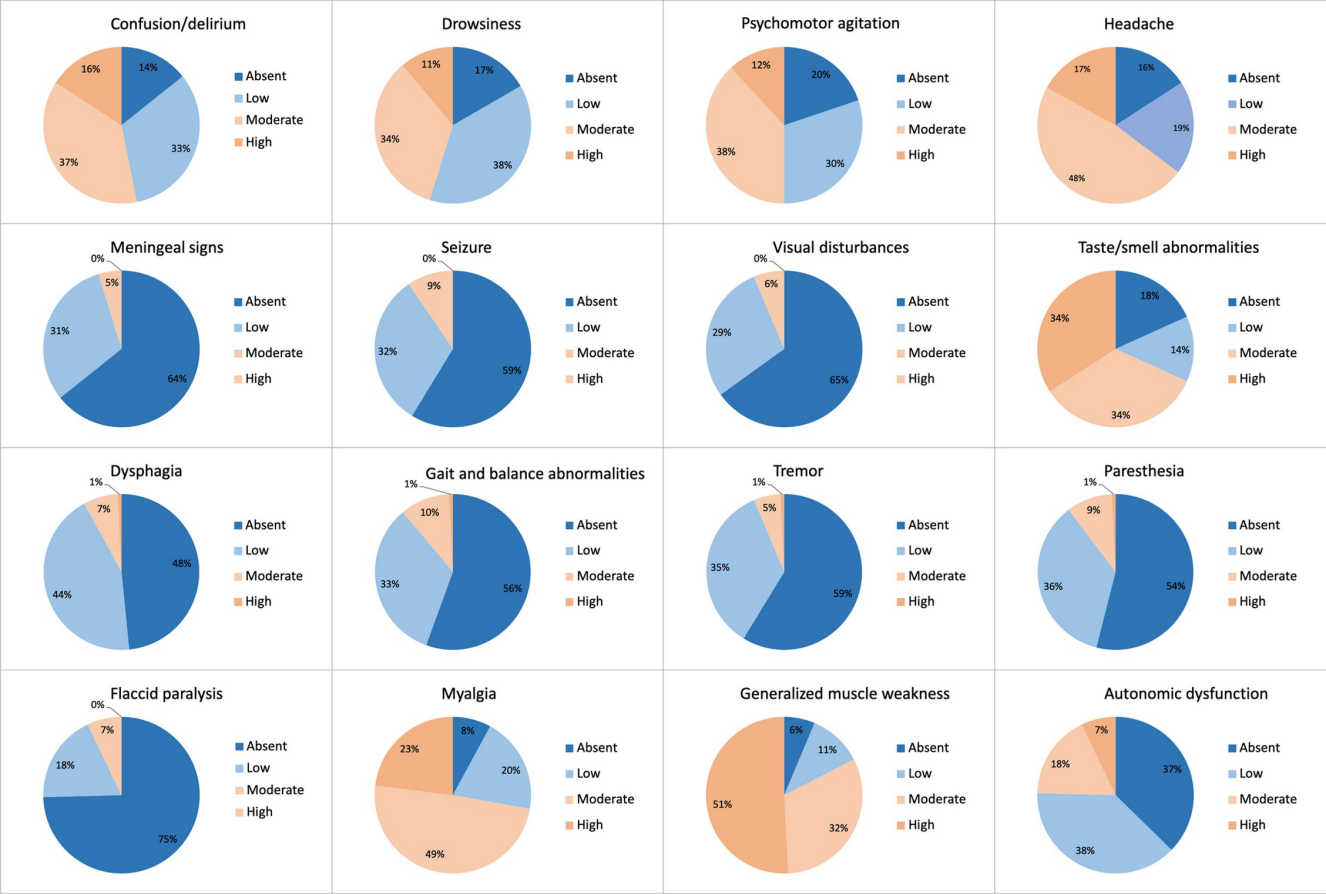
Reply distribution to Question 7: “Evaluate the frequency of these neurological findings in COVID-19 patients”.

Neurological symptoms are reported in 87.3% of the surveys. They are “rare” for 41.2% of doctors, “occasional” for 21.4%, “frequent” for 26.2% and “always present” for 2.4%. Fig 3 describes the symptoms observed (Fig 3). 73.8% of physicians report some correlation between the COVID-19 and neurological symptoms: 41.2% consider neurological symptoms as “probably correlated”, 21.4% with a causal relationship, and 11.1% “incidental”.

Whereas neurologist do not differ from other specialists in detecting neurological symptoms (p = 0.27), “expert” physicians report neurological symptoms with higher frequency than other colleagues (p<0.005). Tables 1 and 2 compare the responses of neurologists and non-neurologist in addressing neurological and non-neurological symptoms and laboratory findings.

**Table 1:** Non neurological symptoms and laboratory findings in neurologists and non-neurologists.

Non neurological symtoms		absent	low	moderate	high	absent (%)	low (%)	moderate (%)	high (%)
Dry Cough	neurologist	2	7	35	33	2.6%	9.1%	45.5%	42.9%
	non neurologist	1	3	17	28	2.0%	6.1%	34.7%	57.1%
Fever	neurologist	2	5	28	42	2.6%	6.5%	36.4%	54.5%
	non neurologist	0	1	6	42	0.0%	2.0%	12.2%	85.7%
Asthenia	neurologist	2	5	26	44	2.6%	6.5%	33.8%	57.1%
	non neurologist	0	3	11	35	0.0%	6.1%	22.4%	71.4%
Dyspnea	neurologist	1	12	43	21	1.3%	15.6%	55.8%	27.3%
	non neurologist	0	8	13	28	0.0%	16.3%	26.5%	57.1%
DIC*	neurologist	19	37	20	1	24.7%	48.1%	26.0%	1.3%
	non neurologist	18	16	14	1	36.7%	32.7%	28.6%	2.0%
Laboratry Findings		absent	low	moderate	high	absent (%)	low (%)	moderate (%)	high (%)
Lymphopenia	neurologist	5	15	28	29	6.5%	19.5%	36.4%	37.7%
	non neurologist	4	5	19	21	8.2%	10.2%	38.8%	42.9%
Lymphocytosis	neurologist	17	37	17	6	22.1%	48.1%	22.1%	7.8%
	non neurologist	18	20	10	1	36.7%	40.8%	20.4%	2.0%
Increased aminotransferase	neurologist	8	19	37	13	10.4%	24.7%	48.1%	16.9%
	non neurologist	5	14	23	7	10.2%	28.6%	46.9%	14.3%
Increased lactate dehydrogenase	neurologist	5	16	30	26	6.5%	20.8%	39.0%	33.8%
	non neurologist	5	9	14	21	10.2%	18.4%	28.6%	42.9%
Increased CPK	neurologist	7	27	34	9	9.1%	35.1%	44.2%	11.7%
	non neurologist	6	13	24	6	12.2%	26.5%	49.0%	12.2%

**Table 2:** Neurological symptoms in neurologists and non-neurologists.

Neurological symtoms		absent	low	moderate	high	absent (%)	low (%)	moderate (%)	high (%)
Confusion/delirium	neurologist	11	20	31	15	14.3%	26.0%	40.3%	19.5%
	non neurologist	7	21	16	5	14.3%	42.9%	32.7%	10.2%
Drowsiness	neurologist	13	26	31	7	16.9%	33.8%	40.3%	9.1%
	non neurologist	8	22	12	7	16.3%	44.9%	24.5%	14.3%
Psychomotor agitation	neurologist	13	24	30	10	16.9%	31.2%	39.0%	13.0%
	non neurologist	12	14	18	5	24.5%	28.6%	36.7%	10.2%
Headache	neurologist	10	17	32	18	13.0%	22.1%	41.6%	23.4%
	non neurologist	6	11	22	10	12.2%	22.4%	44.9%	20.4%
Meningeal signs	neurologist	45	29	3	0	58.4%	37.7%	3.9%	0.0%
	non neurologist	36	10	6	0	73.5%	20.4%	12.2%	0.0%
Seizure	neurologist	36	31	10	0	46.8%	40.3%	13.0%	0.0%
	non neurologist	38	9	2	0	77.6%	18.4%	4.1%	0.0%
Visual symtoms	neurologist	45	24	8	0	58.4%	31.2%	10.4%	0.0%
	non neurologist	37	12	0	0	75.5%	24.5%	0.0%	0.0%
Taste/smell alterations	neurologist	12	7	27	31	15.6%	9.1%	35.1%	40.3%
	non neurologist	11	10	16	12	22.4%	20.4%	32.7%	24.5%
Dysphagia	neurologist	34	39	3	1	44.2%	50.6%	3.9%	1.3%
	non neurologist	27	16	6	0	55.1%	32.7%	12.2%	0.0%
Gait and balance alterations	neurologist	34	32	10	1	44.2%	41.6%	13.0%	1.3%
	non neurologist	36	10	3	0	73.5%	20.4%	6.1%	0.0%
Tremors	neurologist	49	24	4	0	63.6%	31.2%	5.2%	0.0%
	non neurologist	25	20	3	1	51.0%	40.8%	6.1%	2.0%
Paresthesia	neurologist	34	34	9	0	44.2%	44.2%	11.7%	0.0%
	non neurologist	31	11	3	1	12.5%	27.1%	50.0%	12.5%
Flaccid paralysis	neurologist	49	20	8	0	44.2%	44.2%	11.7%	0.0%
	non neurologist	45	3	1	0	12.5%	27.1%	50.0%	12.5%
Myalgia	neurologist	8	18	34	17	44.2%	44.2%	11.7%	0.0%
	non neurologist	2	7	28	12	12.5%	27.1%	50.0%	12.5%
Generalized muscle weakness	neurologist	7	10	23	37	44.2%	44.2%	11.7%	0.0%
	non neurologist	1	4	17	27	12.5%	27.1%	50.0%	12.5%
Autonomic dysfunction	neurologist	25	33	11	8	44.2%	44.2%	11.7%	0.0%
	non neurologist	22	15	11	1	12.5%	27.1%	50.0%	12.5%

## Discussion

This is the first medical survey on the occurrence of neurological symptoms in patients with COVID-19. In a healthcare emergency, an online web-based survey is capable to produce a large amount of data in a short time for a fairly low cost and therefore provides a wide ‘snapshot of how things are at a specific time’ [16]. The main finding is that the majority (87.3%) of physicians involved in the diagnosis and management of acute COVID-19 patients observed neurological symptoms. The most common reported symptoms are headache, myalgias, altered state of conscience and taste and smell abnormalities. Because it is still not clear whether myalgia is caused by inflammation or by direct muscle damage from the virus itself, we decided to include it within neurological symptoms.

Though not directly comparable, our data provides results qualitatively similar to those described in the retrospective analysis by Mao et al. [10]. The pattern of symptoms is similar: headache, myalgias and smell and test abnormalities are the three out of the four most commonly reported disturbances in both studies. Despite the qualitative similarity, they found an overall frequency of neurological symptoms (36.4%) lower than suggested by our survey. The difference could arise from the retrospective design of the study of Mao et al: because the “tip of iceberg” of COVID-19 is the respiratory impairment, neurological symptoms could have been under-reported and/or obscured by more prominent respiratory symptoms. An alternative explanation could be that the virus responsible for the Italian outbreak is different from the one that caused the Chinese disease but there is no demonstration that viral genetic mutations have a clinical impact [17, 18]. Lastly, because the pathogenesis of coronavirus-induced disease is influenced by host genotype [19], the increased occurrence of neurological symptoms could arise -older average age of the European population and a different prevalence of comorbidities aside- from different genetic features of Asiatic and European populations.

Whilst some neurological symptoms reported in the survey can be non-specific, others, such as smell and taste abnormalities, are consistent with the involvement of the central nervous system or cranial nerves. A possible explanation is that SARS-CoV-2 enters into human neurons interacting with angiotensin-converting enzyme 2 (ACE2) receptor expressed also in the brain [20, 21]. Studies in animals demonstrated that SARS-CoV spreads to the cardiorespiratory centers in the brainstem through the olfactory pathway, ultimately leading to death even without major lung involvement [13]. By analogy, also SARS-CoV-2 can be neurotropic and through a central mechanism could contribute to the severe respiratory impairment of COVID-19 patients [4, 13, 22].

Whatever the mechanism, neurological symptoms should be carefully monitored in COVID-19 patients. An important issue will be to assess whether neurological disturbances influence the prognosis of COVID-19.

This study has some limitations: first neurologists and non-neurologists are not represented in equal proportions; second our case number is too small to represent an epidemiology survey of the country. Despite the limited data, we believe that this work provides a comprehensive snapshot of the clinicians’ experience on neurological symptoms of COVID-19.

## Conclusions

87.3% of physicians managing COVID-19 report in their patients the occurrence of neurological symptoms. They can be relevant to understand the pathogenetic mechanism of the disease and develop new treatment strategy. Whilst some of the disturbances reported may be non-specific and common to other infectious diseases, smell and taste abnormalities might indicate nervous system as entry door for SARS-CoV-2 virus. This interpretation should promote research trials to avoid nervous system involvement.

## Supporting information

S1 File Original survey. Survey on neurological symptoms in COVID-19 patients.
(PDF)

S2 File Translated survey. (PDF)

S3 File Original responses. Original responses in sheets.
(XLSX)

S4 File Alberta Research Ethics Community Consensus Initiative (ARECCI) ethic screening tool. (PDF)
