# Supplementary material for: Neurological symptoms in acute COVID-19 infected patients: A survey among Italian physicians
Source: PLoS One. 2020 Sep 11;15(9):e0238159. doi: 10.1371/journal.pone.0238159 (PMC7489055; doi:10.1371/journal.pone.0238159)
Supplement: S1 File — Survey on neurological symptoms in COVID-19 patients. (PDF) [file pone.0238159.s001.pdf]

Questionario sulla presenza di sintomi neurologici nel paziente affetto da SARS-CoV-2

Università degli Studi di Milano
Clinica Neurologica III
Dipartimento di Scienze della Salute
Polo Universitario San Paolo
ASST Santi Paolo e Carlo
Milano

Gentile Collega,
Secondo dati preliminari dei colleghi cinesi (Mao L et al, 2020*), circa il 36% dei pazienti affetti da COVID19 riferisce sintomi neurologici fra i quali alterazioni dello stato di coscienza, disturbi cerebrovascolari e interessamento muscolare. Questi sintomi sarebbero più comuni nei pazienti con form[illegible] severe di malattia ma non è ancora chiaro se siano espressione del quadro sistemico o siano determinati da un coinvolgimento diretto del sistema nervoso. Tale ipotesi potrebbe essere supportata dal fatto che l'invasione del sistema nervoso centrale è documentata per altre forme di coronavirus. Lo studio del coinvolgimento del sistema nervoso potrebbe avere rilevanza nella migliore definizione del quadro clinico. Al fine di impostare uno studio sistematico sull'argomento, ti saremmo grati se volessi compilare questo breve questionario anonimo rivolto ai medici che si stanno confrontando con questi pazienti. Ti saremmo altresì grati se volessi condividerlo con altri colleghi impegnati in questa epidemia.
Il questionario è anonimo ma, nel caso fossi interessato a partecipare alla prosecuzione dello studio, ti invitiamo a segnalarcelo tramite mail (oggetto "NEUROCOVID19") all'indirizzo di posta segr.neurostimolazione@unimi.it. Ti ringraziamo anticipatamente per il tempo che ci vorrai concedere e rimaniamo a tua disposizione per ogni suggerimento.

Prof. Alberto Priori
alberto.priori@unimi.it

Dr. Laura Campiglio
laura.campiglio@asst-santipaolocarlo.it

* Mao L, Wang, M, Chen S, Quanwei H et al. Neurological Manifestations of Hospitalized Patients with COVID-19 in Wuhan, China: A Retrospective Case Series Study (February 24, 2020).
Available at SSRN: https://ssrn.com/abstract=3544840

* Required

1. Area di specializzazione: *

- Medico d'emergenza-urgenza
- Internista
- Pneumologo
- Infettivologo
- Anestesista Rianimatore
- Neurologo
- Cardiologo
- Chirurgo
- Pediatra
- Altro

2. In quale regione esercita? *

- Abruzzo
- Basilicata
- Calabria
- Campania
- Emilia Romagna
- Friuli Venezia e Giulia
- Lazio
- Liguria
- Lombardia
- Marche
- Molise
- Piemonte
- Puglia
- Sardegna
- Sicilia
- Toscana
- Trentino Alto Adige
- Umbria
- Valle d'Aosta
- Veneto

3. Approssimativamente quanti pazienti affetti da COVID-19 ha valutato? *

- < 10
- 10 - 30
- 30 - 50
- > 50

4. Valuti la frequenza di questi sintomi nel paziente affetto da COVID19: *

	assente	bassa	moderata	alta
Tosse secca	☐	☐	☐	☐
Febbre	☐	☐	☐	☐
Astenia	☐	☐	☐	☐
Dispnea	☐	☐	☐	☐
CID	☐	☐	☐	☐

5. Valuti la frequenza di queste alterazioni di laboratorio: *

	assente	bassa	moderata	alta
Linfopenia	☐	☐	☐	☐
Linfocitosi	☐	☐	☐	☐
Rialzo delle transaminasi	☐	☐	☐	☐
Rialzo dell'LDH	☐	☐	☐	☐
Rialzo delle CPK	☐	☐	☐	☐

6. In quanti pazienti affetti da COVID19 ha notato la presenza di sintomi neurologici? *

	1	2	3	4	5	
Nessuno	◯	◯	◯	◯	◯	Tutti

7. Valuti la frequenza dei seguenti sintomi neurologici nei pazienti affetti da COVID19 *

	assente	bassa	moderata	alta
Confusione/delirium	☐	☐	☐	☐
Sopore	☐	☐	☐	☐
Agitazione	☐	☐	☐	☐
Cefalea	☐	☐	☐	☐
Segni meningei	☐	☐	☐	☐
Crisi epilettiche	☐	☐	☐	☐
Disturbi visivi	☐	☐	☐	☐
Alterazioni di gusto/olfatto	☐	☐	☐	☐
Alterazioni Udito	☐	☐	☐	☐
Disfagia	☐	☐	☐	☐
Disturbi di coordinazione/equilibrio	☐	☐	☐	☐
Tremori/spasmi	☐	☐	☐	☐
Parestesie	☐	☐	☐	☐
Paralisi flaccida	☐	☐	☐	☐
Mialgie	☐	☐	☐	☐
Astenia generalizzata	☐	☐	☐	☐
Disautonomia (es.ipotensione, aritmie)	☐	☐	☐	☐

8. Le è capitato di valutare un paziente per sintomatologia neurologica riscontrando in seguit positività per COVID19? *

	1	2	3	4	5	
Mai	◯	◯	◯	◯	◯	Spesso

9. Nel caso si sia presentata l'eventualità descritta nella precedente domanda, ritiene che i sintomi neurologici e l'infezione da COVID19:

- ◯ Non siano correlabili ma coincidenti (es. infezione contratta dopo l'accesso in Ospedale)
- ◯ Siano probabilmente correlabili (es. ipossia e confusione)
- ◯ Vi sia una correlazione causa-effetto fra l'infezione da COVID e i sintomi neurologici
- ◯ Other: ____________________

This content is neither created nor endorsed by Google.

Google Forms
