# Supplementary material for: Neurological symptoms in acute COVID-19 infected patients: A survey among Italian physicians
Source: PLoS One. 2020 Sep 11;15(9):e0238159. doi: 10.1371/journal.pone.0238159 (PMC7489055; doi:10.1371/journal.pone.0238159)
Supplement: S2 File — (PDF) [file pone.0238159.s002.pdf]

Survey on neurological symptoms in COVID-19 patients

Clinical Neurology Unit
University of Milan
Polo Universitario San Paolo
Via Antonio di Rudinì 8,
Milan, 20142 Italy

Dear colleague,

as Covid-19 spreads to pandemic and the number of infected people grows worldwide, questions rise about the potential noxious effect of coronavirus 2 (SARS-Cov-2) on nervous system.
In an early retrospective study Mao et al* reported neurological symptoms in 36% of COVID-19 patients.
It is known that distributions of host receptors are generally consistent with the tropisms of viruses. Previous studies showed that SARS-CoV breaks into human cells interacting with angiotensin-converting enzyme 2 (ACE2) receptor which is expressed in human airway epithelia, lung parenchyma, vascular endothelia, kidney cells, small intestine cells and brain. Experimental studies on transgenic mice expressing human ACE2 demonstrated that SARS-CoV and MERS could spread into CNS via olfactory pathways, causing death without major lung involvement. In such cases, cardiorespiratory center of brainstem was heavily affected. Some authors suggested that also SARS-Cov-2 has neuroinvasive potential and could be implied in the severe respiratory failure observed in COVID19 patients.
In order to quickly assess the involvement of the nervous system we developed an on-line Survey. If you are directly involved in COVID-19 patients care we'll be grateful if you will share your experience with us by filling out the form below. For any question don't hesitate to contact us at the following addresses:

Prof. Alberto Priori
alberto.priori@unimi.it

Dr. Laura Campiglio
laura.campiglio@asst-santipaolocarlo.it

* Mao L, Wang, M, Chen S, Quanwei H et al. Neurological Manifestations of Hospitalized Patients with COVID-19 in Wuhan, China: A Retrospective Case Series Study (February 24, 2020).
Available at SSRN: https://ssrn.com/abstract=3544840

© Campiglio & Priori 2020
* Required

1. Please provide your medical specialty. If you are not specialized, provide your medical experience and other qualifications *

Mark only one oval.

- ALLERGY & IMMUNOLOGY
- ANESTHESIOLOGY
- EMERGENCY MEDICINE
- FAMILY MEDICINE
- INFECTIOUS DISEASE
- INTERNAL MEDICINE
- NEUROLOGY
- PEDIATRICS
- PULMONARY MEDICINE
- SURGERY
- OTHER

2. Please select your region *

3. Approximately how many COVID-19 patients did you evaluated in your clinical practice? *

Mark only one oval.

- < 10
- 10 - 30
- 30 - 50
- > 50

4. Please evaluate the frequency of these symptoms in COVID-19 patients *

Check all that apply.

	Absent	Low	Moderate	High
Fever	☐	☐	☐	☐
Dry cough	☐	☐	☐	☐
Dyspnea	☐	☐	☐	☐
Fatigue	☐	☐	☐	☐
Anorexia	☐	☐	☐	☐
Diarrhea	☐	☐	☐	☐

5. Please evaluate the frequency of these laboratory findings in COVID-19 patients *

Check all that apply.

	Absent	Low	Moderate	High
Lymphopenia	☐	☐	☐	☐
Elevated C-reactive protein	☐	☐	☐	☐
Elevated lactate dehydrogenase	☐	☐	☐	☐
Elevated D-dimer	☐	☐	☐	☐
Elevated liver enzymes	☐	☐	☐	☐
Elevated creatine phosphokinase	☐	☐	☐	☐
Elevated serum creatinine	☐	☐	☐	☐

6. Please estimate the relative frequency of neurological symptoms in COVID-19 patients *

Mark only one oval.

	1	2	3	4	5	
Absent	◯	◯	◯	◯	◯	High

7. Please evaluate the frequency of these symptoms in COVID-19 patients *

Check all that apply.

	Absent	Low	Moderate	High
Headache	☐	☐	☐	☐
Impaired consciousness	☐	☐	☐	☐
Psychomotor agitation	☐	☐	☐	☐
Acute cerebrovascular disease	☐	☐	☐	☐
Ataxia	☐	☐	☐	☐
Epilepsy	☐	☐	☐	☐
Meningeal signs	☐	☐	☐	☐
Visual abnormalities	☐	☐	☐	☐
Taste and smell abnormalities	☐	☐	☐	☐
Dysphagia	☐	☐	☐	☐
Peripheral nerve damage	☐	☐	☐	☐
Myalgias	☐	☐	☐	☐

8. Do you think that the neurological symptoms that you observed were: *

Mark only one oval.

- ( ) Incidental but not related to COVID-19
- ( ) Possibly related to systemic effect of COVID-19 (ex. hypoxia)
- ( ) Directly related to COVID-19 (CNS invasion or neurotropic effect of SARS-CoV-2)

This content is neither created nor endorsed by Google.

Google Forms
