# Supplementary material for: Neurological symptoms in acute COVID-19 infected patients: A survey among Italian physicians
Source: PLoS One. 2020 Sep 11;15(9):e0238159. doi: 10.1371/journal.pone.0238159 (PMC7489055; doi:10.1371/journal.pone.0238159)
Supplement: S4 File — (PDF) [file pone.0238159.s004.pdf]

Alberta Innovates

ARECCI Ethics Screening Tool

Tool Background

Policy or legislative requirements often stipulate that research projects involving people or their health information must be reviewed by a Research Ethics Board (REB). This raises a number of questions. For example, what should be done with projects that are not considered research but involve people or their health information? Should quality improvement (QI) or program evaluation projects also be assessed for their risk to people? What are the characteristics of research versus quality improvement/evaluation projects? How do you decide what to review? How should ethics oversight of these "non-research" projects be approached? Some of these questions remain the subject of lengthy debate.

A pRoject Ethics Community Consensus Initiative (ARECCI) (formerly The Alberta Research Ethics Community Consensus Initiative), an initiative of Alberta Innovates – Health Solutions (AIHS) (formerly the Alberta Heritage Foundation for Medical Research), developed this four-step, web-based ARECCI Ethics Screening Tool to provide practical "on the ground" decision-support assistance to project leaders and teams as they grapple with these very complex questions. Content experts have developed the tool, and its context validity continues to be enhanced through focused implementation with experts and their projects.

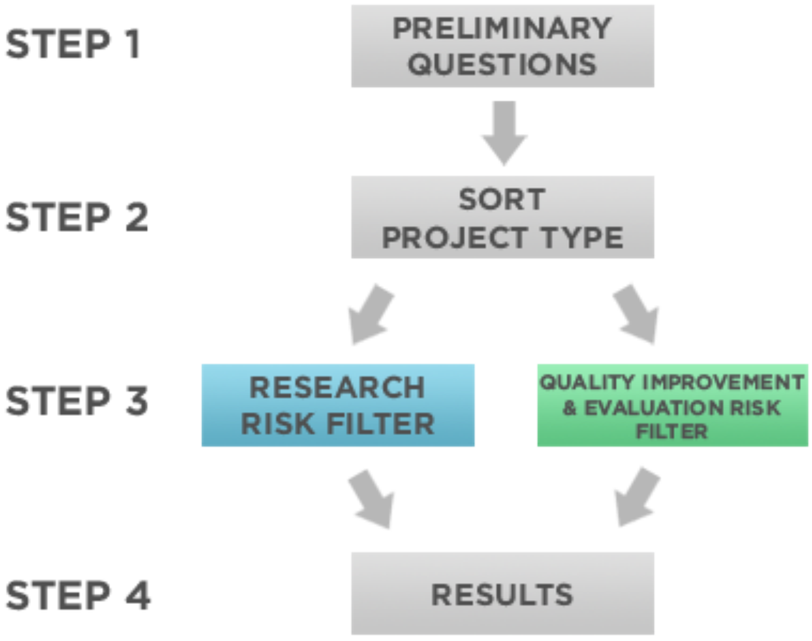

Step 1: PRELIMINARY SCREEN: Helps identify those projects which clearly require REB review.

Step 2: PROJECT PRIMARY PURPOSE: A primary purpose screen sorts research from other types of projects to determine the appropriate review pathway (i.e., REB review or organization/context based oversight).

Step 3: RISK FILTERS: Based on the result in Step 2 (i.e., determination of project primary

purpose), one of two risk filters automatically become available: one for research and one for QI/evaluation. These risk filters help the user identify ethical risks from the perspective of participants in the project.

Step 4: SCREENING RESULT: A summary score produces the category of risk for project participants. The category of risk is highlighted together with the corresponding recommended review action for the project. Specific items and their values that contribute to the total score are also listed. This enables those responsible to plan appropriate risk-mitigating strategies before involving participants. Professional judgment is required in interpreting all screening results.

Email, Save, Print, and Notes functions have been incorporated throughout the tool to assist project team discussion and planning.

Legend

Provides more information about a question (if available).

Save the current state of the tool and retrieve a URL for bookmarking.
Note: This is not the same as selecting File > Save As in your browser. Please use this save function instead.

E-mail the results of the tool.

Print the results of the tool.

Submit feedback regarding the tool.

Process

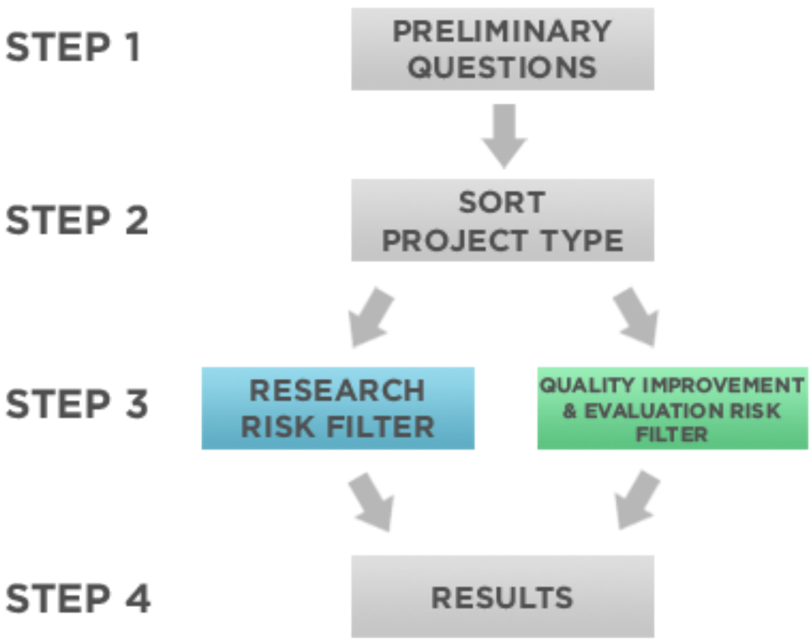

Project Title:

Date Completed: August 07, 2020

Neurological symptoms in acute COVID-19 infected patients: a survey among Italian physicians.

Step 1: Preliminary Questions

1. Is there an explicit requirement for review of this project by a Research Ethics Board as part of its funding arrangements? Yes ○ No ●

This item refers to projects where the funder requires ethics review by a REB. Examples of such funding agencies are: the Canadian Institutes of Health Research, the Canadian Health Services Research Foundation, the Natural Sciences and Engineering Research Council of Canada, the Social Sciences and Humanities Research Council, and Alberta Innovates – Health Solutions. Projects funded by these agencies are typically (but not always) considered research and all are required to undergo REB ethics review.

2. Are there any local policies that require this project to undergo review by a Research Ethics Board? Yes ○ No ●

The intent of this item is to allow flexibility in the tool for different organizational local policies (where they exist) regarding requirements for research ethics board review. For example, some jurisdictions require that all student projects must undergo ethics review by a designated REB, regardless of project classification.

3. Does the project involve use of a pharmaceutical device, drug or natural health product under Health Canada Food and Drug Act regulations or guidelines? Yes ○ No ●

Under the Health Canada Food and Drug Act regulations or guidelines, REB review is required. For more information please see: http://www.hc-sc.gc.ca/dhp-mps/legislation/index-eng.php NOTE: This applies to the development of a new device, drug, or natural health product or the testing of any of these for a use different from the original approval by Health Canada. This item does not apply to understanding or improving the use of an approved product in the local context.

Notes:

Step 2: About Your Project

4. Is the project designed to test a specific hypothesis or answer a specific quantitative or qualitative question? Yes ◯ No ◉

This question helps assess whether your project fits in one of the two broad research approaches: quantitative and qualitative. A key component in this item is assessing whether or not there is a clearly stated research question. Qualitative research projects are guided by specifically formulated research questions. These types of research projects apply explicit qualitative theory which underlie and direct the methodology used in the design of the specific study, including the analysis plan. Quantitative research projects are directed by specific hypotheses or research questions that guide the selection of the scientific design of the specific study, including the analysis methods. In general, qualitative research develops theory through rigor in interpretation of observations. In general, quantitative research tests theory through the measurement of key variables.

5. Does the project involve a comparison of control groups? Yes ◯ No ◉

This question helps determine if your project fits a research design that uses multiple groups or sites to “control” for unrelated factors in the study. "Control" is considered important for rigor (precision) in studying the key variables of focus in this type of project. Projects designed to include such scientific control follow internationally accepted standards related to how they are going to be conducted with features such as precise power calculations and other techniques.

6. Is the project designed to support generalizations that go beyond the particular population the sample is being drawn from? Yes ◉ No ◯

This question assesses whether the design of your project fits with research that is specifically designed to produce results that can be assumed to be true (generalized) beyond the individual participants in the specific study. In other words, with the clear intent of following internationally accepted scientific standards for "generalizability", your project design includes precise sample size calculations and other techniques related to how it is going to be conducted. Research designed for “generalizability” implies some future application of findings to the population of focus, although sometimes subjects do directly benefit from participation in a research project. Note: Producing and sharing learnings from a project for potential adaptation to other contexts is not the same thing as producing results that are considered scientifically generalizable because of specific features included in the design of the study such as precise power calculations.

7. Does the project impose any additional burdens on participants beyond what would be normally expected or normally experienced during the course of care, program participation or role expectations? Yes ◯ No ◉

This question helps determine your project's fit with research in that participation is voluntary and that those participating will be involved in activities which are in addition to routine care, program provision, or other routine actions or duties on the part of the participant.

8. Is the primary purpose of the project to produce the kind of results that could be published in a research journal? Yes ◉ No ◯

This question clarifies whether the main goal of your project is to obtain results that CAN be published in a research type of journal. In other words, the most important reason you are doing this study is to contribute to the general body of knowledge on the topic through achieving scientific publication. By contrast, the main goal in quality improvement and evaluation is to provide information for decisions about a specific program or aspect of service delivery. Sharing by publication is a secondary goal in these non-research projects.

9. Will project participants also likely be among those who might potentially benefit from the result of the project as it proceeds? Yes ○ No ●

This question helps determine your projects fit with quality improvement or evaluation. Quality improvement and evaluation projects provide timely and specific feedback on a program or process in a particular organization, setting, program or service. Thus, participants are more likely to benefit from findings produced in these projects than are subjects in research projects.

10. Is the project intended to develop a better practice within your organization or setting? Yes ● No ○

This question clarifies if the main goal of your project is to produce findings that can be used to improve practice, program or service delivery within your organization or setting. In other words, the most important reason you are doing this study is to contribute in a timely manner to improving how some aspect of care or service is delivered in a particular location.

11. Would this project still be done at your site even if the results might not be applicable anywhere else? Yes ○ No ●

This question helps assess if your project fits with the usual focus of quality improvement and evaluation on site-specific programs, services or processes. By contrast, in research the specific site does not matter except in more general terms such as urban or rural. Please note, in the due course of time you may choose to share (through presentation at conferences or publication in an Evaluation or QI journal) the process and results of your project with others for adaptation to new contexts. However, sharing project results for potential benefit elsewhere is not the main reason you are doing the project.

12. Does the language used in the project description refer specifically to features of a particular program, organization, or locale, rather than using more general terminology such as rural vs. urban populations? Yes ○ No ●

The language used in your project can help determine if it is quality improvement/ evaluation or research. Quality improvement and evaluation projects use terminology that specifically name a particular program or process, or a particular organization, setting, or service. By contrast, research projects often describe location by more general characteristics such as rural versus urban, which reflects their intent to be "generalizable" across settings.

13. Is the current project part of a continuous process of gathering or monitoring data within an organization? Yes ○ No ●

This question helps to assess the fit of your project with the primary focus of quality

improvement. The focus of QI is on time-limited projects that target service, program, or process improvements. QI projects are often initiated in response to issues and trends identified through ongoing quality assurance monitoring of care and service provision.

Notes:

Your score indicates that the most probable purpose of your project is Research.
Please proceed to determine the category of risk to your participants.

Does your project involve...

14. Collection of data through physically or clinically invasive procedures? Yes ○ No ◉

15. Collection of data through non-invasive procedures involving imaging or microwaves? Yes ○ No ◉

16. Collection, use, or disclosure of health information, biological samples, or other personal or private information where the researcher is requesting that the requirement for informed consent be waived? Yes ○ No ◉

Informed consent is a requirement for all human subject research; however, there are circumstances where such consent may be difficult to obtain. The TCPS addresses such circumstances in Article 2.1 and requires that the investigator apply to a REB for approval to waive the requirement for informed consent prior to implementation of any aspect of the project.

17. Procedures related to anaesthetics or sedation not normally required for participant care? Yes ○ No ◉

18. Deception or intended incomplete disclosure of the nature of the Yes ○ No ◉

study?

19. Likelihood that a breach of confidentiality could place participants at risk of legal liability, denial of insurance or other damage to financial standing, employability or reputation? Yes ○ No ●

There is widespread agreement about the rights of research participants to privacy and the corresponding duty of investigators to treat private information in a respectful and confidential manner (TCPS p. 3.1). This item assesses whether the current project is higher risk with respect to the protection of privacy and the consequences for the participant should confidentiality of that private information be breached.
While the best protection of the confidentiality of personal information and records is through anonymity, when that is not possible project leaders should indicate the extent of the confidentiality that can be promised to participants and the countermeasures that are put in place to mitigate (ease the response should it occur) this risk. These should be clearly outlined on the consent form and during the consent process, including a plan to limit access to and provide secure storage of the private information for a specified period of time and with a specific plan for its destruction at the end of that timeframe as appropriate.

20. Questions or procedures that might cause participants psychological distress, discomfort or anxiety beyond what a reasonable person might expect in day-to-day interactions? Yes ○ No ●

For example, questions that raise painful memories or unresolved emotional issues or procedures that involve manipulation is some manner may be anticipated to potentially cause discomfort, anxiety or distress in participants. Project leaders should anticipate all potential reactions that may be triggered by such questions or procedures, and include counter measures designed to minimize (reduce or curtail the magnitude of the potential response) or mitigate (ease the response should it occur) these reactions in project participants. Appropriately trained personnel administering the questions or procedures and providing support and resource contact information, are but a few of such countermeasures. The consent form needs to include any potential risks that participants may be exposed to and describe how these will be minimized or mitigated.

21. Questions that involve sensitive issues such as sexual orientation or practices, illegal behaviour, stigmatizing conditions or diagnoses, religious or cultural beliefs or practices? Yes ○ No ●

Questions that touch on these and other sensitive issues may be anticipated to potentially cause participants to be cautious in how they respond. This private information once collected may have consequences beyond the project that need to be anticipated in advance. Countermeasures to protect privacy and confidentiality which minimize or mitigate any potential negative impacts on participants should be built into the plan. The consent form needs to include any potential risk that participants may be exposed to as well as outline the planned countermeasures. Countermeasures can include: appropriately trained personnel to collect the information, linkage to appropriate support resources, and a solid plan for access to and secure transmission / storage of personally identifiable data.

22. A power relationship between the investigator and participants (e.g., manager/employee, therapist/client, teacher/student)? Yes ○ No ●

This risk has to do with the requirement in a research project for informed consent to be freely given with the ability to freely withdraw at any time. The TCPS states that the element of voluntariness has important implications for how freely and informed consent may given or withdrawn by participants if undue influence is present by virtue of existing relationships in the institutional context in which the project will be carried out (see Article 2.2). This undue influence may restrict participants in how freely they can give consent or withdraw consent. This arises when the elements of trust and dependency are present in relationships such as manager/ employee, health provider/patient and teacher/student. In projects where this risk may arise its design has to include countermeasures that reduce any "form of inducement, deprivation or exercise of control or authority over prospective subjects". In the case of the manager/employee situation, suggestions include having someone else as project lead and data collector with all data collected anonymized to the respective manager of the employees. All risks and counter measures should be clearly outlined in the consent form.

23. A real or potential conflict of interest between an investigator and the sponsor of the investigation? Yes ○ No ◉

Any conflict of interest of this nature needs to be declared upfront and measures put in place to counteract any real or potential undue influence on any aspect of the project including data collection, analysis and reporting of findings.

24. Blood and tissue samples for genetic/DNA testing or storage for future research purposes? Yes ○ No ◉

This risk has to do with the requirement in a research project that the use of tissue depends on the individual's altruism in donating with the expectation that social good will be advanced. The TCPS Section 10 provides guidance that continuing consent and/or free and informed consent concerning new research projects have to be clearly addressed. In the case of genetic research an added dimension is that the tissue may reveal information about one's current or future health and that of biological relatives (Section 10.1). It is essential to ensure protection of the privacy of the individual, confidentiality of their information and appropriate informed consent through ethics review by a REB.

25. Collection of blood sample volumes exceeding (i) and (ii) below? healthy, non-pregnant adults weighing at least 50 kg (amounts drawn may not exceed 550 ml in an eight week period, and collection cannot occur more often than twice per week); from other adults and children if the amount drawn does not exceed the lesser of 50 ml or 3 ml per kg in an eight week period (collection cannot occur more often than twice per week); Yes ○ No ◉

26. Therapeutic procedures in clinical trials that are themselves known to pose considerable risks of harm (e.g., surgery, chemotherapy, radiation therapy)? Yes ○ No ◉

27. Clinical studies on drugs and medical devices when an investigational device exemption application or investigational new drug application is not required (e.g., if it is a non-invasive diagnostic device) or if the medical device, drug or natural health product has been cleared or approved for marketing for that purpose/indication? Yes ○ No ◉

28. Special populations or any individuals or groups in a socially vulnerable position? Yes ○ No ●

Special populations include but are not limited to pregnant women, children, frail elderly, prisoners, refugee claimants, students, and staff. Examples of individual behaviours that may contribute to vulnerability include but are not limited to perception, cognition, motivation, identity, language, communication, social behaviour and cultural beliefs or practices. The TCPS states that ethical obligations to vulnerable individuals and populations often require special procedures to protect their interests (p i.5).

29. Use of personally identifiable data, documents, records or specimens originally collected for therapeutic purposes? Yes ○ No ●

Data on individuals originally collected as part of routine care, program participation or role expectations and which is identifiable cannot then just be used for research purposes. Clinicians or other providers who have ready access to such data by virtue of their role and who propose to do such research first require ethics approval by a REB before implementing any aspect of their project. Projects which propose to use such identifiable data (e.g., chart reviews) must adhere to the secondary use of data guidelines outlined in the TCPS in Articles 3.4 to 3.5, and the appropriate articles in the respective Health Information Act of their province, if the information collected is health related.

30. Collection of data from voice, video, digital or image recordings? Yes ○ No ●

There is risk that using these methods to collect private information from participants may have the potential to breach their confidentiality by revealing personally identifying information. The consent form should include this risk and outline countermeasures to protect the privacy of individuals and their information. To mitigate and minimize this risk, a well thought out plan needs to be in place regarding the secure storage of this private data and who has access to it.

31. The use of tests, survey procedures, interview procedures, oral history, focus groups or observation of public behaviour where the participants can be identified directly or indirectly through the information recorded? Yes ○ No ●

Tests can include but are not limited to cognitive, diagnostic, achievement, and aptitude. The risk here is that using these methods to collect private information from research participants may have the potential to breach their confidentiality by revealing personal information. The consent form needs to include any potential risks that participants may be exposed to and outline the planned counter measures. Appropriately trained personnel to collect the information and appropriate plans for access and secure storage of the private data are also important components of the plan.

32. Prospective collection of biological specimens for research purposes by non-invasive means (e.g., hair and nail clippings, mucosal and skin cells collected by buccal swab, skin swab or mouth washings)? Yes ○ No ●

Prospective refers to data which will be collected in the future compared to retrospective which refers to data which has been collected in the past.

33. Collection of data through non-invasive procedures routinely Yes ○ No ●

employed in clinical settings?

Examples of non-invasive procedures routinely used in clinical care include In BP, Ht, Wt, and TPR readings.

34. Student research projects? Yes ◯ No ◉

There may be potential for greater risk in projects where students are involved because students can sometimes lack the experience or skills needed to carry out research projects and may potentially in some cases not have the supervision needed to overcome these lacks which can increase the risk to participants.

Notes:

Your score is 0. The project involves Minimal Risk. Use the ARECCI tools to identify and manage risk consistent with local policies.

Ethics Screening Score Cutoff Points

Score Result	Level (Category) of Risk	Recommended Ethics Review
15 or greater	Definitely greater than minimal	Full review* consistent with local policies
8 - 14	Somewhat more than minimal	Seek a second opinion regarding screening result to further explore review requirements
+ 0 - 7	Minimal	Delegated* review consistent with local policies

+ There is always potential for ethical risk in projects that involve people or their personal information.

*The term "delegated" replaces "expedited" according to the Tri-Council Policy Statement

(TCPS) [Canadian Institutes of Health Research, National Sciences and Engineering Research Council of Canada and the Social Sciences and Research Council of Canada. 2005. Tri-Council Policy Statement on Research Involving Human Subjects. Ottawa, ON: Interagency Secretariat on Research Ethics.]

If you are unsure about any of the above, please consult a knowledgeable source about your project.

Feedback is welcomed on this tool- please use the feedback link to the right.

For more information, please feel free to contact:
Alberta Innovates
Suite 1500, 10104 - 103 ave
Edmonton AB T5J 0H8
Email: ARECCI.health@albertainnovates.ca Tel: 780.423.5727
